# Problematic Internet Use and Family Rules Among Children and Adolescents

**DOI:** 10.1002/npr2.70155

**Published:** 2026-07-15

**Authors:** Saya Moriyama, Takayuki Harada

**Affiliations:** ^1^ Doctoral Program in Counseling Science, Graduate School of Comprehensive Human Sciences, Degree Programs in Comprehensive Human Sciences University of Tsukuba Tsukuba Japan; ^2^ Institute of Human Sciences University of Tsukuba Tsukuba Japan

**Keywords:** adolescent, family, parents, prevention, problematic internet use

## Abstract

This study investigates the relationship between problematic Internet use (PIU) and family rules regarding Internet use among children and adolescents. Although the Internet offers valuable development opportunities, excessive use is associated with adverse outcomes. Family‐related factors have been discussed in relation to PIU prevention; however, empirical evidence regarding the establishment and maintenance of family rules remains limited. Data were obtained from a large‐scale administrative survey conducted in Japan, including 8534 elementary and junior high school students. PIU was assessed using the Young Diagnostic Questionnaire. Family rules were classified according to their presence, the rule‐setting process (parents only, parents and children collaboratively, or children), and the degree of adherence. Associations between family rules and PIU were examined using logistic regression analyses adjusted for sex, grade, and time spent on the Internet on weekdays and holidays. The results indicated that having previously established but no longer maintained family rules was associated with higher odds of PIU. In contrast, collaboratively established rules between parents and children were associated with lower odds of PIU, whereas rules determined by parents alone were associated with higher odds of PIU. Additionally, lower adherence to family rules was associated with higher odds of PIU. Collectively, these findings suggest that not only the presence of family rules but also the process through which they are established and adhered to are associated with PIU among children and adolescents. Accordingly, collaborative rule‐setting and rule adherence may be relevant family processes to consider in future longitudinal and intervention research on PIU.

## Abstract 2

Among 8534 Japanese children and adolescents, previously established but no longer maintained family Internet rules were linked to higher odds of problematic Internet use (PIU). Collaboratively set rules were associated with lower PIU odds, whereas parent‐only rules and low rule adherence were linked to higher odds.

## Graphical abstract

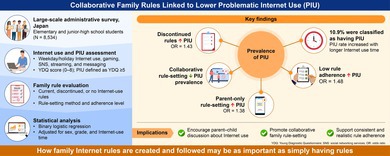

## 1 Introduction

The Internet is increasingly used for entertainment, socialization, education, and information acquisition. According to a survey by Japan's Children and Families Agency [1], 98.2% of children between the ages of 10 and 17 use the Internet for various purposes, including hobbies, entertainment, communication, and study. The literature shows that the Internet supports the individual development of children and adolescents by providing access to information, supportive communities, and spaces for self‐expression [2, 3]. However, excessive Internet use is associated with several negative consequences among children and adolescents, including sleep disturbance [4], reduced academic achievement [5], and deterioration of their psychosocial well‐being [6].

Young proposed the idea of Internet addiction (IA) [7], and screening tests such as the Young's Diagnostic Questionnaire have been developed. In addition to IA, terms such as “problematic Internet use,” “compulsive Internet use,” and “pathological Internet use” have been used to refer to patterns of problematic behavior associated with excessive Internet use [4, 8]. Regarding online games, gaming disorder was officially added as a condition in the International Classification of Diseases‐11 [9] and is a condition requiring further study in DSM‐5‐TR [10]. Problematic use is observed not only for online games but also for other services, such as social networking services and streaming [11, 12]. Although uniform terminology has not been established, the risks of using the Internet are increasingly accepted. In this study, we used the term “problematic internet use” (PIU), which has been widely used in previous studies, as a generic term for excessive and compulsive Internet use [13, 14, 15].

Although variations exist among countries and regions, a review reported an estimated prevalence of PIU of 6% in 31 nations across seven world regions [16]. In particular, the prevalence of PIU among adolescents aged 19 years or younger is higher than that in young and elderly adults [17]; moreover, several studies reported that PIU can be observed from early adolescence and peaks at the age of 15–16 years [18]. These reports suggest that adolescents may be particularly vulnerable to PIU. PIU has been reported among elementary school children [19, 20], highlighting the importance of early attention to PIU for the healthy development of children.

PIU is often discussed within the broader context of behavioral addictions because it involves features such as impaired control, salience, and continued use despite negative consequences, which overlap with those observed in Gaming Disorder [9, 10]. Adolescents may be particularly vulnerable to such behavioral problems because self‐regulatory capacities are still developing during this period. The prefrontal cortex, which is involved in inhibitory control, decision‐making, and self‐regulation, continues to mature into early adulthood, while reward‐related systems develop relatively earlier [21]. From this developmental perspective, family‐related guidance and structure may play an important role in supporting children's and adolescents' regulation of recreational Internet use.

Building on this developmental perspective, previous literature has emphasized the importance of family‐related factors in PIU among children and adolescents [22, 23]. Factors such as parent–child closeness [24], family functioning [25, 26], and family activities [27] have been associated with the development of PIU, with lower levels linked to higher PIU. On the other hand, parental involvement in Internet use is considered one of the most important protective factors [28]. However, empirical findings regarding this factor are inconsistent. Low parental involvement has been associated with higher PIU [29], whereas other studies have reported protective associations with parental supervision [30, 31]. In contrast, several studies have demonstrated that restrictive parental involvement is positively associated with PIU [26, 32]. Furthermore, cross‐sectional studies have suggested that strict parental rules regarding time spent online may be associated with higher levels of PIU [32] and that increasing the number and strictness of rules may be associated with a higher likelihood of PIU [26].

Overall, these inconsistent findings underscore that focusing solely on the presence or strictness of parental control may insufficiently capture the complexity of family processes linked to PIU. Specifically, prior research has rarely examined how family rules regarding Internet use are established, such as whether they are determined unilaterally by parents or collaboratively through parent–child discussions, or whether children actually adhere to such rules. From a family‐process perspective, the manner in which rules are negotiated and maintained may be as important as the rules themselves. Although screen time restrictions are often argued to be more effective when parents and children share mutual agreement [33], this assumption has rarely been tested empirically. Therefore, this study extends previous research by distinguishing between how rules are established and how consistently they are followed, and by examining their associations with PIU among children and adolescents.

## 2 Method

### 2.1 Participants and Procedures

Study data were obtained from the Fuchu City Survey on Internet Use Among Youth, conducted by the City of Fuchu, an urban municipality in Tokyo, Japan. The survey—targeting 12 693 public school students from the third grade of elementary school to the third grade of junior high school in Fuchu City as well as their parents or guardians—aimed to assess Internet use patterns, related behaviors, and associated lifestyle factors among children and adolescents. Data were collected using an online questionnaire administered between April 11 and May 9, 2022. Overall, 10 298 students participated in the survey.

This study analyzed data from 8534 children and adolescents, ranging from third‐grade elementary school to third‐grade junior high school, who reported regular Internet use. The analytical sample comprised 4344 male students, 3965 female students, and 225 participants who did not report their sex. Although the survey also targeted parents or guardians and the parental questionnaire included items assessing family Internet‐use rules, parent and child responses were collected through separate channels: parents responded via a city‐administered email system, whereas children responded via their schools. Because no linking information was collected, individual‐level linkage of parent and child questionnaires was not possible. In addition, the parental questionnaire did not include an assessment of PIU. Therefore, the present analyses were restricted to data provided by children and adolescents.

The survey was commissioned by the City of Fuchu and implemented by an external research firm on behalf of the municipality, with the primary purpose of informing local policy and preventive measures related to Internet use among the youth. This study utilized fully anonymized individual‐level data from this survey for secondary analysis. No personally identifiable information (e.g., names and contact details) was provided to the researchers, and individual respondents could not be re‐identified from the dataset used in this study.

The dataset analyzed in this study is not publicly available. However, detailed information and an official overview of the Fuchu City Survey on Internet Use Among Youth are available on the official website (in Japanese): https://www.city.fuchu.tokyo.jp/kosodate/kyoiku/ikuse/seishounen_net_chousa.html.

### 2.2 Measures

#### 2.2.1 Sociodemographic and Internet Use Behavior

Sociodemographic data regarding participants' grade and sex were collected. Moreover, the assessment of Internet‐use behavior included determining participants' daily time spent (i.e., number of hours) on the Internet for non‐study purposes on weekdays and holidays. In addition, participants were asked to indicate the types of Internet activities they engaged in, including gaming, social networking services, streaming/video, novel/manga reading, information search, email, blog/forum use, chat/messaging, and other services. These activity variables were treated as binary indicators in the supplementary analyses.

#### 2.2.2 Problematic Internet Use

The Young Diagnostic Questionnaire (YDQ) is a self‐report scale comprising eight dichotomous (“yes” or “no”) items, with total scores ranging from 0 to 8. A response of “yes” is scored as 1 and “no” as 0. Young proposed that respondents who answered “yes” to five or more items could be classified as having Internet addiction [7]. The YDQ has been widely used in previous studies and, because of its small number of items, is considered an easy and feasible tool for large‐scale surveys. The Japanese version of the YDQ [34] was back‐translated to minimize language bias. In the present sample, the internal consistency was moderate (Cronbach's α = 0.67), while the observed value was within the range reported in previous large‐scale studies using the YDQ (approximately 0.65–0.75) [35, 36, 37]. Students with YDQ scores ranging from 0 to 4 were classified into the Normal Internet Use (NIU) group, whereas those with scores ranging from 5 to 8 were classified into the PIU group.

#### 2.2.3 Family Rules Regarding Internet Use

The participants were asked to choose from four response alternatives regarding family rules for Internet use (“With rules,” “There used to be, but not now,” “Never decided,” and “Don't know”). Participants who answered “With rules” were subsequently asked how the rules had been established, with the following three response alternatives: “Determined by parents,” indicating that rules were decided primarily by parents without explicit discussion with the child; “Determined by parents and children,” indicating that rules were established through discussion or agreement between parents and children; and “Determined by children,” indicating that rules were decided primarily by the child. Additionally, adherence to the rules was assessed using a 5‐point scale ranging from 1 (“mostly followed”) to 5 (“almost never followed”), with higher scores indicating lower adherence to the rules.

### 2.3 Statistical Analysis

All statistical analyses were conducted using SPSS (version 30.0), and statistical significance was set at a *p* < 0.05. Categorical variables were summarized as numbers and proportions. Cases with missing data on variables included in each analysis were excluded using listwise deletion. Differences between the NIU and PIU groups were examined using chi‐squared tests for categorical variables.

Binary logistic regression analyses were conducted to examine the associations between family rules regarding Internet use and PIU. Two models were estimated. In Model 1, PIU (NIU = 0, PIU = 1) was entered as the dependent variable, and family rules regarding Internet use were entered as the independent variable. Sex, grade, and time spent on the Internet on weekdays and holidays were included as covariates.

In Model 2, analyses were restricted to participants who reported having current family rules regarding Internet use. PIU was entered as the dependent variable, and the method of rule setting (parents only, parents and children, or children only) and the degree of adherence to the rules were entered as independent variables. Sex, grade, and time spent on the Internet on weekdays and holidays were included as covariates.

Time spent on the Internet on weekdays and holidays was originally assessed using categorical response alternatives representing ranges of daily use for non‐study purposes. For the logistic regression analyses, these variables were treated as continuous by assigning each category's midpoint. Categorical variables were entered using dummy coding, with reference categories specified in each model. The degree of adherence to the rules was treated as an ordinal variable, with higher scores indicating lower adherence. Adjusted odds ratios (ORs) and 95% confidence intervals (CIs) were calculated. Sex, grade, and time spent on the Internet were included as covariates because prior epidemiological studies have reported that these factors are associated with PIU [4, 29, 35].

To address potential effect modification by developmental stage and to assess the robustness of the main findings, supplementary analyses were conducted. Interaction terms between school level (elementary, grades 3 to 6; junior high, grades 7 to 9) and each family‐rule variable were tested in logistic regression models to examine whether associations differed by developmental stage. Where statistically significant interactions were observed, stratified models were estimated separately for elementary and junior‐high school students. Cross‐tabulations of family‐rule categories by grade were also performed to characterize sample distribution. Finally, to address the possibility that potential confounding by Internet activity type might confound the associations between family‐rule variables and PIU, Models 1 and 2 were additionally adjusted for the nine Internet activity indicators described above. Detailed results of these supplementary analyses are presented in Tables S1–S5.

## 3 Results

### 3.1 Descriptive Statistics

Table 1 shows the demographics of the individuals in the NIU and PIU groups. In total, 928 (10.9%) participants (*n* = 8534) were classified as the PIU group. The PIU rate was significantly higher for female students than for male students (*p* < 0.001). Regarding school grades, the rate of PIU differed significantly across grades (*p* < 0.001). The highest rate was observed among sixth‐grade students (14.2%), whereas the lowest rate was observed among seventh‐grade students (7.3%). The rate of PIU increased significantly with greater time spent on the Internet on both weekdays and holidays (*p* < 0.001). Regarding family rules about Internet use, the proportion of participants classified as PIU differed significantly across response categories (*p* < 0.001), with the highest rate observed among those who reported “Used to be, but not now” (18.8%).

**TABLE 1:** Sex, school grade, family rules on Internet use, and daily average time of Internet use, based on scores on the Young Diagnostic Questionnaire (YDQ).

	Total	NIU^a^ group	PIU^b^ group	
	*N* = 8534	*N* = 7606	*N* = 928	
	*N* (%)	*N* (%)	*N* (%)	Statistics
Sex				*χ* ^*2*^(2) = 15.29, *p* < 0.001
Male	4344 (100.0)	3918 (90.2)	426 (9.8)	
Female	3965 (100.0)	3500 (88.3)	465 (11.7)	
Other	225 (100.0)	188 (83.6)	37 (16.4)	
Grade				*χ* ^*2*^(6) = 34.33, *P* < 0.001
3rd	849 (100.0)	769 (90.6)	80 (9.4)	
4th	981 (100.0)	883 (90.0)	98 (10.0)	
5th	1078 (100.0)	957 (88.8)	121 (11.2)	
6th	1193 (100.0)	1024 (85.8)	169 (14.2)	
7th	1212 (100.0)	1124 (92.7)	88 (7.3)	
8th	1562 (100.0)	1387 (88.8)	175 (11.2)	
9th	1659 (100.0)	1462 (88.1)	197 (11.9)	
Time spent on the Internet on weekdays (hours)				*χ* ^*2*^(7) = 360.37, *P* < 0.001
Nothing	206 (100.0)	195 (94.7)	11 (5.3)	
< 1	1393 (100.0)	1333 (95.7)	60 (4.3)	
1–2	2869 (100.0)	2660 (92.7)	209 (7.3)	
2–3	1763 (100.0)	1573 (89.2)	190 (10.8)	
3–4	1038 (100.0)	876 (84.4)	162 (15.6)	
4–5	503 (100.0)	412 (81.9)	91 (18.1)	
5–6	288 (100.0)	214 (74.3)	74 (25.7)	
≥ 6	474 (100.0)	343 (72.4)	131 (27.6)	
Time spent on the Internet on holidays (hours)				*χ* ^*2*^(7) = 400.96, *P* < 0.001
Nothing	166 (100.0)	160 (96.4)	6 (3.6)	
< 1	810 (100.0)	782 (96.5)	28 (3.5)	
1–2	1763 (100.0)	1679 (95.2)	84 (4.8)	
2–3	1624 (100.0)	1503 (92.5)	121 (7.5)	
3–4	1190 (100.0)	1071 (90.0)	119 (10.0)	
4–5	818 (100.0)	701 (85.7)	117 (14.3)	
5–6	683 (100.0)	571 (83.6)	112 (16.4)	
≥ 6	1480 (100.0)	1139 (77.0)	341 (23.0)	
Family rules regarding Internet use				*χ* ^*2*^(3) = 62.38, *P* < 0.001
With rules	5573 (100.0)	5033 (90.3)	540 (9.7)	
There used to be, but not now	799 (100.0)	649 (81.2)	150 (18.8)	
Never decided	722 (100.0)	654 (90.6)	68 (9.4)	
Don't know	1440 (100.0)	1270 (88.2)	170 (11.8)	

^a^ NIU, Normal Internet use (YDQ score ≤ 4 points).

^b^ PIU: Problematic Internet use (YDQ score ≥ 5 points).

### 3.2 Family Rules and PIU

Of the participants (*n* = 5573), 65.3% indicated that they had family rules regarding Internet use. Table 2 compares the NIU and PIU groups regarding how the rules were set and whether they were followed. The results revealed a relationship between how the rules were set and PIU. Based on standardized residuals, those who reported only parental rules had a higher PIU rate (z = 5.66, *p <* 0.01), whereas those who reported that the rules were set by the parents and children had a lower PIU rate (z = −5.66, *p <* 0.01). Regarding the degree to which the rules were followed, the “Mostly followed” group had a significantly lower percentage of PIU compared with the overall distribution across adherence categories (z = 14.27, *p <* 0.01). The percentage of PIU increased as adherence to the rules decreased.

**TABLE 2:** How the family rules were set and whether they were followed.

	Total (with rules)	NIU^a^ group	PIU^b^ group	Statistics
	*N* = 5573	*N* = 5033	*N* = 540	
	*N* (%)	*N* (%)	*N* (%)	
How to determine rules				
By parents and children	2819 (100.0)	2605 (92.4)	214 (7.6)	*χ* ^*2*^(2) = 32.09, *p* < 0.001
By parents	2617 (100.0)	2301 (87.9)	316 (12.1)	
By children	137 (100.0)	127 (92.7)	10 (7.3)	
Degree of adherence to the rules				
Mostly	3686 (100.0)	3478 (94.4)	208 (5.6)	*χ* ^*2*^(4) = 239.27, *p* < 0.001
Sometimes	853 (100.0)	732 (85.8)	121 (14.2)	
Neither	506 (100.0)	421 (83.2)	85 (16.8)	
Rarely	353 (100.0)	270 (76.5)	83 (23.5)	
Almost never	175 (100.0)	132 (75.4)	43 (24.6)	

^a^ NIU, Normal Internet use (YDQ score ≤ 4 points).

^b^ PIU, Problematic Internet use (YDQ score ≥ 5 points).

### 3.3 Associations Between Family Rules Regarding Internet Use and PIU

Table 3 presents the results of binary logistic regression analyses examining the associations between family‐rule variables and PIU.

**TABLE 3:** Association between the YDQ categorical groups (PIU vs. NIU) and each variable: Results of binary logistic regression analyses.

Model 1 (*N* = 8,534)			
Variables	Adjusted OR	95% CI	*p*
Family rules regarding Internet use			
With rules	Reference		
There used to be, but not now	1.43	1.16–1.77	0.001
Never decided	0.63	0.49–0.86	0.001
Don’t know	1.00	0.82–1.21	0.99
Sex			
Male	Reference		
Female	1.32	1.15–1.53	< 0.001
Other	1.63	1.10–2.40	0.01
Grade			
3rd	Reference		
4th	1.03	0.75–1.42	0.85
5th	1.06	0.78–1.44	0.72
6th	1.30	0.97–1.74	0.08
7th	0.56	0.40–0.78	0.001
8th	0.76	0.57–1.02	0.07
9th	0.89	0.67–1.12	0.43
Time spent on the Internet on weekdays (hours)	1.04	0.99–1.10	0.08
Time spent on the Internet on holidays (hours)	1.25	1.20–1.29	< 0.001

Model 2 (*N* = 5,573)			
Variables	Adjusted OR	95% CI	*p*
How to set rules			
By parents and children	Reference		
By parents	1.38	1.14–1.67	0.001
By children	0.80	0.40–1.60	0.53
Degree of adherence to the rules	1.48	1.38–1.59	< 0.001
Sex			
Male	Reference		
Female	1.23	1.01–1.49	0.04
Other	2.01	1.27–3.38	0.004
Grade			
3rd	Reference		
4th	0.78	0.51–1.20	0.26
5th	0.91	0.61–1.36	0.65
6th	1.12	0.76–1.64	0.58
7th	0.47	0.31–0.72	< 0.001
8th	0.69	0.47–1.02	0.06
9th	0.83	0.57–1.20	0.33
Time spent on the Internet on weekdays (hours)	1.01	0.94–1.09	0.79
Time spent on the Internet on holidays (hours)	1.23	1.17–1.30	< 0.001

Abbreviations: CI, Confidence interval; OR, Odds ratio.

In Model 1, participants who responded “Used to be, but not now” exhibited higher odds of PIU (adjusted OR = 1.43; 95% CI: 1.16–1.77, *p* = 0.001) than those who reported having current family rules (reference category). The model included sex, grade, and time spent on the Internet on weekdays and holidays, and the association remained significant after adjusting for these covariates. Conversely, participants who reported that rules were “never set” exhibited lower odds of PIU (adjusted OR = 0.63; 95% CI: 0.49–0.86, *p* = 0.001).

In Model 2, participants who responded that the rules were set by the parents alone had higher odds of PIU (adjusted OR = 1.38; 95% CI: 1.14–1.67, *p* = 0.001). Moreover, lower adherence to the rules was significantly associated with higher odds of PIU (adjusted OR = 1.48; 95% CI: 1.38–1.59, *p* < 0.001).

Additional analyses examined whether the associations between family‐rule variables and PIU differed by school level. Significant interactions with school level were observed for rule presence (*p* < 0.001), rule‐setting method (*p* = 0.001), and adherence to the rules (*p* < 0.001; Table S4). In stratified analyses, the association between the “Used to be, but not now” category and higher odds of PIU was more pronounced among elementary‐school students (adjusted OR = 1.91; 95% CI: 1.37–2.65) than among junior‐high students (adjusted OR = 1.16; 95% CI: 0.87–1.54). Conversely, the lower odds associated with the “Never decided” category were observed primarily among junior‐high students (adjusted OR = 0.52; 95% CI: 0.35–0.78), but not among elementary‐school students (adjusted OR = 0.75; 95% CI: 0.50–1.12; Table S2). Parent‐only rule‐setting and lower adherence to the rules were associated with higher odds of PIU in both school‐level groups, although the magnitude of the adherence association was somewhat greater among elementary‐school students (Table S3). The “Used to be, but not now” category was more prevalent in higher grades, particularly eighth and ninth grade (Supplementary Table S1).

To address the possibility that the type of Internet activity might confound the observed associations, Models 1 and 2 were further adjusted for nine self‐reported activity categories. The associations between family‐rule variables and PIU remained essentially unchanged after this adjustment (Table S5). For example, the adjusted ORs for the “Used to be, but not now” category, parent‐only rule‐setting, and adherence to the rules were 1.37, 1.40, and 1.46, respectively. Among the activity categories, streaming/video use showed the strongest association with PIU in the fully adjusted models; however, this finding was secondary to the main focus of the present study and should therefore be interpreted with caution.

## 4 Discussion

This study reported associations between PIU and family rules regarding Internet use. Importantly, this study extends prior research by distinguishing not only the presence or absence of family rules, but also the process by which such rules were established and the degree to which they were adhered to. By simultaneously examining rule‐setting processes and rule adherence within a large community‐based sample, the present study provides a more refined understanding of family dynamics related to PIU than previous studies that have primarily focused on the strictness or existence of parental control.

A higher percentage of PIU was observed among children who reported that family rules regarding Internet use had existed previously but were no longer in place at the time of the survey. Stratified analyses, however, suggested that this association differed by school level: the association was more pronounced among elementary‐school students than among junior‐high students. Moreover, the “Used to be, but not now” group was disproportionately concentrated in higher grades.

Given the cross‐sectional design of the study, the abovementioned association should not be interpreted as evidence that discontinuation of family rules contributes to PIU or that maintaining rules prevents PIU. The observed pattern is consistent with at least two complementary interpretations. First, the association may partly reflect reverse causality: children and adolescents with higher levels of PIU may have previously prompted parents to establish rules, which may have been weakened, abandoned, or perceived as ineffective over time. Second, the “Used to be, but not now” category may also reflect normative developmental transitions during adolescence, including increasing autonomy and corresponding reductions in direct parental control [38]. The concentration of this group in higher grades is compatible with this developmental interpretation. The stronger association observed among elementary‐school students may suggest that this rule‐status category is more closely related to PIU among younger children, whose self‐regulatory capacities are still developing.

This developmental pattern provides a theoretical context for interpreting the findings. The prefrontal cortex, which is involved in inhibitory control and decision‐making, continues to mature throughout adolescence and is particularly immature in early adolescence [21]. Self‐regulatory capacities also develop gradually through interactions with parents and other caregivers, and parenting behaviors that provide structure and guidance have been linked to children's self‐regulatory development [39, 40]. In this context, consistent family rules may reflect one form of external structure or guidance related to children's developing self‐regulation of recreational Internet use. This view is also broadly consistent with previous findings showing that adolescents who perceived some degree of control over their Internet use had lower levels of PIU [41]. However, because of the cross‐sectional design, the present study cannot determine whether family rules precede or follow PIU.

In contrast to expectations, the absence of family rules (i.e., the “Never decided” category) was associated with lower odds of PIU compared with having current rules. Stratified analyses further showed that this association was present primarily in junior‐high students and was not statistically significant in elementary‐school students. This developmental pattern is consistent with a possible rule‐necessity bias, whereby families without explicit Internet rules may include adolescents who already show relatively stable patterns of Internet use or higher self‐regulatory capacities, particularly in junior high school, when self‐regulatory capacities generally become more developed. In this case, the absence of rules may reflect the consequence of low‐risk Internet use rather than functioning as a protective factor. Among younger children, whose self‐regulatory capacities are still emerging, the absence of rules may not be similarly correlated with already‐established self‐regulation, which may explain why this association was not observed in the elementary‐school subsample. Family factors that have been associated with lower PIU, such as positive parent–child relationships or parent–child closeness, may also partly account for the lower PIU observed in the absence of rules [24, 42]. Because these factors were not directly measured in the present study, the underlying mechanisms remain unclear, and longitudinal research is needed to disentangle the directionality.

Regarding how rules are decided among participants with current family rules, parent‐only rule‐setting was associated with higher odds of PIU than decisions made through cooperation between parents and children. A previous study investigating the relationship between smartphone addiction and parental control in children showed that increased parental control was associated with increased smartphone addiction [43]. Another study reported a positive association between parental rules regarding time spent online and PIU [32]. To the extent that parent‐only rule‐setting may be associated with a more restrictive approach to parental mediation, the present findings are broadly consistent with these earlier reports. By contrast, previous research has demonstrated that adolescents who have satisfactory conversations with their parents regarding Internet use showed lower levels of PIU [32]. Collectively, these findings suggest that the process of rule‐setting—rather than the mere presence of rules—may serve as a proxy for underlying family processes related to PIU. However, because the rule‐setting variable captured only who determined the rules and did not directly assess communication quality or the quality of the parent–child relationship, these results should not be interpreted as direct evidence of specific family processes. An alternative, child‐driven explanation is also possible: parents may be more likely to set rules unilaterally when children exhibit behavioral difficulties or other individual characteristics that themselves independently increase the risk of PIU. Because the present data cannot distinguish between parent‐driven and child‐driven explanations for this association, future research should examine characteristics of both parents and children as potential moderators. Similar associations for parent‐only rule‐setting were observed in both elementary‐ and junior‐high‐school students, and the estimates were essentially unchanged after additional adjustment for Internet activity type. These findings suggest that the association between rule‐setting process and PIU is not solely attributable to school level or differences in Internet activity type.

Lower adherence to the rules was associated with higher odds of PIU. The association was somewhat stronger among elementary‐school students than among junior‐high students, although it remained substantial in both subgroups. Because difficulty in controlling time and frequency of Internet use is a core feature of PIU [44], the cross‐sectional design of this study does not allow us to determine whether lower adherence precedes PIU or represents a behavioral correlate or manifestation of PIU itself. In the latter case, supportive approaches that address underlying patterns of compulsive use may be more appropriate than simply tightening restrictions. Longitudinal research is needed to distinguish these possibilities and to clarify the role of rule adherence in the development and maintenance of PIU.

This study has several limitations. The cross‐sectional design precludes inferences regarding temporal or causal relationships. The observed associations may reflect bidirectional processes, reverse causality, rule‐necessity bias, or unmeasured factors affecting both family‐rule practices and PIU. Longitudinal studies are needed to clarify the temporal sequence among rule‐setting processes, rule adherence, and PIU. Although the overall sample was large, some family‐rule categories were unevenly distributed, and estimates for smaller subgroups, particularly the “By children” category, should be interpreted with caution. The internal consistency of the Japanese YDQ was moderate (Cronbach's α = 0.67), although comparable to values reported in previous epidemiological studies using the YDQ [35, 36, 37]. This may have introduced classification error and affected the precision of the estimated associations. The study relied exclusively on self‐reported data from children and adolescents. The original survey targeted both students and their parents or guardians, and the parental questionnaire included items assessing family Internet‐use rules. However, parent and child responses were collected through separate channels, and no linking information was collected. Therefore, individual‐level linkage of parent and child questionnaires was not possible. In addition, the parental questionnaire did not assess PIU. As a result, parent–child concordance in perceptions of family rules could not be examined, nor could parental perceptions be used as a complementary measure of the child‐reported family‐rule variables. Future research should incorporate parental reports, teacher assessments, and objective indicators of Internet use. Several potentially important confounding variables were not measured in the present study. These include family functioning, parent–child relationship quality, socioeconomic status, and child characteristics such as ADHD or ASD traits and broader psychiatric status. These factors may influence both how family rules are established and the likelihood of PIU, and their omission limits the conclusions that can be drawn from the current findings. Although Internet activity type was additionally adjusted for, activity measures were limited to binary indicators and did not capture frequency, duration, or specific applications used. Finally, the findings may not generalize to private‐school students, rural populations, or students not attending school at the time of the survey, including those with more severe PIU.

## 5 Conclusions

This study examined the associations between family rules regarding Internet use and PIU among elementary and junior high school students in Japan. The results showed that PIU was more prevalent among children who reported having had family rules in the past but not at present. Regarding how the rules were established, collaboratively established rules were associated with lower PIU prevalence than parent‐only rule‐setting. In addition, lower adherence to established rules was associated with higher PIU prevalence. Future longitudinal and intervention studies are needed to clarify the temporal relationships among rule‐setting processes, rule adherence, and PIU, and to examine how family‐based approaches can support healthy Internet use among children and adolescents.

## Author Contributions

S.M.: Conceptualization; Methodology; Formal analysis; Investigation; Writing – original draft; Writing – review and editing; Supervision. T.H.: Writing – review and editing; Supervision.

## Funding

The authors have nothing to report.

## Ethics Statement

This study was based on data from the “Fuchu City Survey on Internet Use Among Youth” conducted by the City of Fuchu, Tokyo, Japan. The original survey was commissioned by the municipal government and implemented by KENZAN Inc., a research company with which the first author was affiliated at the time of data collection. The first author was involved in conducting the survey but was not involved in any policy decision‐making processes associated with the survey findings. This study, which involved a secondary analysis of fully anonymized administrative survey data, was conducted as part of the author's doctoral research at the University of Tsukuba. The dataset utilized for this study contained no personally identifiable information, and individual respondents could not be identified from the data. The secondary analysis—including study design, statistical analyses, interpretation of results, and manuscript preparation—was conducted independently by the author and was not part of the municipal contract. This study was conducted in accordance with the academic guidelines and research governance procedures of the University of Tsukuba and complied with the ethical principles outlined in the Declaration of Helsinki, particularly regarding the protection of participant confidentiality and responsible use of research data. According to Japanese regulations, including the Act on the Protection of Personal Information and the Ethical Guidelines for Life Science and Medical Research Involving Human Subjects issued by the Ministry of Health, Labor, and Welfare, the secondary analysis of fully anonymized administrative data does not require prior approval from an institutional review board or ethics committee. Therefore, formal ethical approval was not required for this study.

## Consent

Consent was obtained by the municipal government from participants/guardians at the time of the original survey. Participants and, where applicable, their parents or guardians were informed of the survey purpose, the voluntary nature of participation, and the handling of data, including secondary use for research purposes. This study involved a secondary analysis of fully anonymized data, and no additional consent was required for this analysis.

## Conflicts of Interest

The authors declare no conflicts of interest.

## Supporting information


**Table S1:** Cross‐tabulation of family‐rule categories by grade.
**Table S2:** Stratified Model 1 by school level (elementary vs. junior high).
**Table S3:** Stratified Model 2 by school level (elementary vs. junior high).
**Table S4:** Interaction tests between school level and family‐rule variables.
**Table S5:** Model 1 and Model 2 with additional adjustment for the content of Internet activity.

## Data Availability

The dataset analyzed in this study is not publicly available because of data‐use agreements with the City of Fuchu. However, detailed information and an official overview of the Fuchu City Survey on Internet Use Among the Youth are available on Fuchu's official website (in Japanese): https://www.city.fuchu.tokyo.jp/kosodate/kyoiku/ikuse/seishounen_net_chousa.html.
