# Supplementary material for: Problematic Internet Use and Family Rules Among Children and Adolescents
Source: Neuropsychopharmacol Rep. 2026 Jul 15;46(3):e70155. doi: 10.1002/npr2.70155 (PMC13373444; doi:10.1002/npr2.70155)
Supplement: Supplementary file 1 — Table S1: Cross‐tabulation of family‐rule categories by grade. Table S2: Stratified Model 1 by school level (elementary vs. junior high). Table S3: Stratified Model 2 by school level (elementary vs. junior high). Table S4: Interaction tests between school level and family‐rule variables. Table S5: Model 1 and Model 2 with additional adjustment for the content of Internet activity. [file NPR2-46-e70155-s001.docx]

**Supplementary Material**

Problematic Internet Use and Family Rules Among Children and Adolescents

This document contains five Supplementary Tables (S1 to S5) reporting the additional analyses described in the Methods section. Detailed results of the interaction tests, stratified analyses by school level, cross-tabulation of family-rule categories by grade, and models adjusted for the content of Internet activity are provided below.

**Contents:**

Supplementary Table S1. Cross-tabulation of family-rule categories by grade.

Supplementary Table S2. Stratified Model 1 by school level (elementary vs. junior high).

Supplementary Table S3. Stratified Model 2 by school level (elementary vs. junior high).

Supplementary Table S4. Interaction tests between school level and family-rule variables.

Supplementary Table S5. Model 1 and Model 2 with additional adjustment for the content of Internet activity.

**Supplementary Table S1**

*Cross-tabulation of family-rule categories by grade (N = 8,534).*

| **Family-rule category** | **G3** | **G4** | **G5** | **G6** | **G7** | **G8** | **G9** | **Total** |
| --- | --- | --- | --- | --- | --- | --- | --- | --- |
| With rules | 477 | 615 | 750 | 807 | 889 | 968 | 1,067 | 5,573 |
| Row % | 8.6 | 11.0 | 13.5 | 14.5 | 16.0 | 17.4 | 19.1 | 100.0 |
| Grade % | 56.2 | 62.7 | 69.6 | 67.6 | 73.3 | 62.0 | 64.3 | 65.3 |
| Std. residual | −5.9 | −1.8 | +3.2 | +1.8 | +6.4 | −3.1 | −0.9 |  |
| Used to be, but not now | 53 | 62 | 74 | 78 | 117 | 190 | 225 | 799 |
| Row % | 6.6 | 7.8 | 9.3 | 9.8 | 14.6 | 23.8 | 28.2 | 100.0 |
| Grade % | 6.2 | 6.3 | 6.9 | 6.5 | 9.7 | 12.2 | 13.6 | 9.4 |
| Std. residual | −3.3 | −3.5 | −3.0 | −3.6 | +0.4 | +4.2 | +6.5 |  |
| Never decided | 90 | 81 | 62 | 94 | 73 | 156 | 166 | 722 |
| Row % | 12.5 | 11.2 | 8.6 | 13.0 | 10.1 | 21.6 | 23.0 | 100.0 |
| Grade % | 10.6 | 8.3 | 5.8 | 7.9 | 6.0 | 10.0 | 10.0 | 8.5 |
| Std. residual | +2.4 | −0.2 | −3.4 | −0.8 | −3.3 | +2.4 | +2.5 |  |
| Don't know | 229 | 223 | 192 | 214 | 133 | 248 | 201 | 1,440 |
| Row % | 15.9 | 15.5 | 13.3 | 14.9 | 9.2 | 17.2 | 14.0 | 100.0 |
| Grade % | 27.0 | 22.7 | 17.8 | 17.9 | 11.0 | 15.9 | 12.1 | 16.9 |
| Std. residual | +8.3 | +5.2 | +0.9 | +1.1 | −5.9 | −1.2 | −5.8 |  |
| Total (Grade) | 849 | 981 | 1,078 | 1,193 | 1,212 | 1,562 | 1,659 | 8,534 |

*Note:* G3 to G9 = grades 3 (third grade of elementary school) to 9 (third grade of junior high school). Row % = percentage within each family-rule category; Grade % = percentage within each grade. Standardized residuals greater than ±2.0 indicate notable deviations from the expected count under independence. The chi-square test for independence was significant (*χ*² = 388.4, *df* = 18, *P* < 0.001). The "Used to be, but not now" category was disproportionately concentrated in eighth and ninth grade (standardized residuals +4.2 and +6.5).

**Supplementary Table S2**

*Stratified Model 1: Associations between family-rule presence and PIU by school level.*

| **Variable** | **Elementary**  **(N = 4,101)** |  | **Junior High**  **(N = 4,433)** |  |
| --- | --- | --- | --- | --- |
|  | aOR (95% CI) | *P* | aOR (95% CI) | *P* |
| Family rules regarding Internet use |  |  |  |  |
| With rules | Reference |  | Reference |  |
| Used to be, but not now | 1.91 (1.37–2.65) | <0.001 | 1.16 (0.87–1.54) | 0.313 |
| Never decided | 0.75 (0.50–1.12) | 0.161 | 0.52 (0.35–0.78) | 0.001 |
| Don't know | 1.16 (0.91–1.50) | 0.232 | 0.82 (0.60–1.11) | 0.195 |
| Sex |  |  |  |  |
| Male | Reference |  | Reference |  |
| Female | 1.25 (1.02–1.54) | 0.030 | 1.39 (1.13–1.70) | 0.002 |
| Other | 1.19 (0.63–2.24) | 0.591 | 1.97 (1.20–3.24) | 0.008 |
| Time on Internet on weekdays (hours) | 1.04 (0.97–1.11) | 0.258 | 1.05 (0.98–1.13) | 0.164 |
| Time on Internet on holidays (hours) | 1.23 (1.17–1.30) | <0.001 | 1.26 (1.20–1.34) | <0.001 |

*Note*: aOR = adjusted odds ratio; CI = confidence interval. Models were stratified by school level: elementary (grades 3 to 6) and junior high (grades 7 to 9). Grade was included as a covariate within each subsample (estimates for individual grade dummies omitted for brevity). Reference category for the family-rule variable is "With rules."

**Supplementary Table S3**

*Stratified Model 2: Associations between rule-setting method, adherence, and PIU by school level (participants with current family rules).*

| **Variable** | **Elementary**  **(N = 2,649)** |  | **Junior High**  **(N = 2,924)** |  |
| --- | --- | --- | --- | --- |
|  | aOR (95% CI) | *P* | aOR (95% CI) | *P* |
| Rule-setting method |  |  |  |  |
| By parents and children | Reference |  | Reference |  |
| By parents only | 1.40 (1.06–1.85) | 0.017 | 1.35 (1.04–1.77) | 0.027 |
| By children only | 1.22 (0.56–2.63) | 0.617 | 0.20 (0.03–1.49) | 0.115 |
| Adherence to rules (1=mostly to 5=almost never) | 1.58 (1.42–1.75) | <0.001 | 1.40 (1.27–1.54) | <0.001 |
| Sex |  |  |  |  |
| Male | Reference |  | Reference |  |
| Female | 1.21 (0.92–1.59) | 0.179 | 1.28 (0.98–1.67) | 0.074 |
| Other | 0.99 (0.36–2.69) | 0.979 | 2.96 (1.65–5.30) | <0.001 |
| Time on Internet on weekdays (hours) | 1.01 (0.92–1.11) | 0.823 | 1.00 (0.89–1.11) | 0.960 |
| Time on Internet on holidays (hours) | 1.23 (1.14–1.32) | <0.001 | 1.24 (1.15–1.34) | <0.001 |

*Note*: aOR = adjusted odds ratio; CI = confidence interval. Models were restricted to participants with current family rules and stratified by school level. Grade was included as a covariate within each subsample (estimates for individual grade dummies omitted for brevity). Reference category for rule-setting method is "By parents and children." Adherence was treated as a continuous ordinal variable, with higher values indicating lower adherence.

**Supplementary Table S4**

*Interaction tests between school level and family-rule variables.*

| **Interaction** | **Sample** | **Score χ²** | **df** | ***P* (Score)** |
| --- | --- | --- | --- | --- |
| Rule presence × School level (Model 1) | N = 8,534 | 18.0 | 3 | <0.001 |
| Adherence × School level (Model 2) | N = 5,573 | 44.6 | 1 | <0.001 |
| Rule-setting method × School level (Model 2) | N = 5,573 | 13.0 | 2 | 0.001 |

*Note*: Interaction terms between school level (elementary = grades 3 to 6 vs. junior high = grades 7 to 9) and each family-rule variable were tested in binary logistic regression. Each model included sex, grade, weekday and holiday Internet use time, and the relevant family-rule variable as covariates. Score tests are reported because Wald tests can lose statistical power under cell-size imbalance. Significant interactions indicate that the association between the family-rule variable and PIU differed by school level, motivating the stratified analyses presented in Supplementary Tables S2 and S3.

**Supplementary Table S5**

*Model 1 and Model 2 with additional adjustment for the content of Internet activity.*

**Panel A. Model 1 (N = 8,534)**

| **Variable** | **Original aOR**  **(95% CI)** | **Activity-adjusted aOR**  **(95% CI)** | ***P* (adjusted)** |
| --- | --- | --- | --- |
| Family rules regarding Internet use |  |  |  |
| With rules | Reference | Reference |  |
| Used to be, but not now | 1.43 (1.16–1.77) | 1.37 (1.10–1.69) | 0.005 |
| Never decided | 0.63 (0.49–0.86) | 0.61 (0.46–0.81) | <0.001 |
| Don't know | 1.00 (0.82–1.21) | 1.02 (0.84–1.24) | 0.808 |
| Internet activity (Yes vs. No) |  |  |  |
| Gaming | — | 1.21 (1.00–1.46) | 0.046 |
| Social networking services | — | 1.04 (0.86–1.25) | 0.717 |
| Streaming / video | — | 1.91 (1.42–2.57) | <0.001 |
| Novel / manga | — | 1.29 (1.09–1.53) | 0.003 |
| Information search | — | 0.81 (0.69–0.96) | 0.015 |
| Email | — | 1.04 (0.88–1.22) | 0.620 |
| Blog / forum | — | 1.62 (1.27–2.09) | <0.001 |
| Chat / messaging | — | 1.26 (1.02–1.56) | 0.033 |
| Other services | — | 1.08 (0.70–1.67) | 0.732 |

*Note*: "Original aOR" refers to estimates from the Model 1 presented in Table 3, without adjustment for the content of Internet activity. "Activity-adjusted aOR" refers to estimates after additional adjustment for the nine binary activity indicators. Both models also adjusted for sex, grade, and weekday and holiday Internet use time (estimates not shown). The associations between family-rule variables and PIU remained essentially unchanged after activity adjustment.

**Panel B. Model 2 (N = 5,573, participants with current family rules)**

| **Variable** | **Original aOR**  **(95% CI)** | **Activity-adjusted aOR**  **(95% CI)** | ***P* (adjusted)** |
| --- | --- | --- | --- |
| Rule-setting method |  |  |  |
| By parents and children | Reference | Reference |  |
| By parents only | 1.38 (1.14–1.67) | 1.40 (1.16–1.70) | <0.001 |
| By children only | 0.80 (0.40–1.60) | 0.78 (0.39–1.56) | 0.479 |
| Adherence to rules | 1.48 (1.38–1.59) | 1.46 (1.36–1.57) | <0.001 |
| Internet activity (Yes vs. No) |  |  |  |
| Gaming | — | 1.15 (0.91–1.46) | 0.246 |
| Social networking services | — | 0.98 (0.77–1.26) | 0.880 |
| Streaming / video | — | 2.07 (1.40–3.07) | <0.001 |
| Novel / manga | — | 1.24 (1.00–1.55) | 0.054 |
| Information search | — | 0.74 (0.60–0.92) | 0.007 |
| Email | — | 1.18 (0.97–1.44) | 0.107 |
| Blog / forum | — | 1.32 (0.93–1.87) | 0.118 |
| Chat / messaging | — | 1.31 (0.98–1.75) | 0.071 |
| Other services | — | 1.16 (0.67–2.00) | 0.593 |

*Note*: "Original aOR" refers to estimates from the Model 2 presented in Table 3, without adjustment for the content of Internet activity. "Activity-adjusted aOR" refers to estimates after additional adjustment for the nine binary activity indicators. Both models also adjusted for sex, grade, and weekday and holiday Internet use time (estimates not shown). Adherence was treated as a continuous ordinal variable, with higher values indicating lower adherence. The associations between rule-setting method, adherence, and PIU remained essentially unchanged after activity adjustment.
